# COVID-19 Related Media Consumption and Mental Health in Older Adults

**DOI:** 10.1093/geroni/igab046.3581

**Published:** 2021-12-17

**Authors:** Erta Cenko, Christopher Kaufmann, Todd Manini

**Affiliations:** 1 University of Florida, Gainesville, Florida, United States; 2 University of Florida

## Abstract

At the beginning of the COVID-19 pandemic, consuming media was critical to identify precautionary behaviors to reduce the spread of the virus, particularly for older adults. Media consumption leads to heightened awareness, but may also negatively affect mental health. We examined whether non-social and social media consumption impacted anxiety and depression relative to pre-COVID-19 symptoms. We conducted an anonymous, cross-sectional survey in May and June 2020. Participants (n=1,168, 73.2 years, 56.8% women, 94.9% White), were asked to estimate their amount of time spent consuming pandemic-related media each day, and to report on anxiety and depressive symptoms both before and after the pandemic onset. We characterized change in anxiety and depression by subtracting scores on current anxiety and depressive symptoms from their recalled symptoms prior to the pandemic. Respondents with high pandemic-related media consumption (>3hrs) were more likely to have increased anxiety, compared to those with low (<1hr) media consumption (OR:1.57, 95%CI:1.09-2.23). Similarly, respondents with increased social media consumption during the pandemic were 64% more likely to have depression, compared to those who did not use social media. This association was bi-directional— those who reduced their social media use were 45% less likely to have depression and 26% less likely to have anxiety, compared to those who never used social media. Older adults consuming more pandemic-related media had increased anxiety. Increased social media consumption was associated with elevated depression symptoms. The potential benefits of media consumption about the COVID-19 pandemic may have unintended negative consequences on mental health.

